# Seroprevalence of Chagas disease in urban and rural indigenous populations of the south of Gran Chaco

**DOI:** 10.1590/0037-8682-0479-2021

**Published:** 2022-04-29

**Authors:** Carlina Colussi, Mariana Stafuza, Marcelo Nepote, Diego Mendicino

**Affiliations:** 1 Universidad Nacional del Litoral, Facultad de Bioquímica y Ciencias Biológicas, Centro de Investigaciones sobre Endemias Nacionales. Santa Fe, Argentina.; 2 Ministerio de Salud de la Provincia de Santa Fe, Hospital Central de Reconquista. Reconquista, Argentina.; 3 Ministerio de Salud de la Provincia de Santa Fe, Programa Provincial de Control de la Enfermedad de Chagas. Santa Fe, Argentina.; 4 Consejo de Investigaciones Científicas y Técnicas. Buenos Aires, Argentina.

**Keywords:** Trypanosoma cruzi, Chagas disease, Seroprevalence, Indigenous populations, Urbanization.

## Abstract

**Background:**

In Latin America, Chagas disease is endemic, with a high prevalence in rural indigenous communities and an increasing prevalence in urban areas owing to migration from rural areas with active vector transmission. This study aimed to assess differences in the prevalence of Chagas disease in urban and rural moqoit communities, one of the main ethnic indigenous groups in the south of Gran Chaco.

**Methods:**

A seroprevalence study was conducted in six moqoit populations in the Santa Fe province, Argentina. The variables studied were serology results for Chagas disease, residents of urban or rural areas, age, and sex.

**Results:**

The results showed that 9.26% of the 702 volunteers evaluated and 18.32% of the 131women of childbearing potential were seropositive. According to the calculated prevalence ratio, the prevalence of Chagas disease in urban communities was6.41 (95% confidence inverval: 3.73-11.02) times higher than that in rural communities: 21.59% in urban communities vs. 3.37%in rural communities.

**Conclusions:**

The seroprevalence found in the moqoit community was higher than the estimated level for the general population of the same region, with a greater impact in urban areas than in rural areas. The urbanization of groups of people with poor socio-sanitary conditions in the second half of the 20th century could have caused this higher seroprevalence of Chagas disease.

## INTRODUCTION

Chagas disease is a parasitic infection caused by the protozoan,*Trypanosoma cruzi*. It is transmitted either through triatomine hematophagous insects (vector pathway) or congenitally, transfusionally, by organ transplantation, laboratory accidents with *Trypanosoma cruzi* cultures, or by intake of contaminated food (non-vector pathways)^1^. 

Triatomines that transmit the infection are exclusive to Latin America, with a greater presence in rural areas of the Gran Chaco eco-region^2^. This eco-region is characterized by a subtropical climate with a large thermal amplitude and high summer temperatures. It is also characterized by a xerophytic forest crossed by plain rivers, low population density, scattered precarious housing, poor socioeconomic indicators, and a large proportion of the population made up of indigenous communities^3^.

In the south of Gran Chaco, the prevalence of Chagas disease is high in rural indigenous communities in the presence of triatomines in their homes^4^ ^,^ ^5^, and has increased in urban areas due to migration from areas with active vector transmission (Chagas disease urbanization) due to unfavorable socio-sanitary conditions^6^ ^-^ ^8^. Due to regional efforts, the Santa Fe province was certified as free of vector transmission by the Pan American Health Organization in 2012.

The main factors associated with Chagas disease in urban centers include migration from an endemic areawith vector transmission^9^ or descent from mothers with these risk factors, due to the possibility of congenital transmission^10^. Currently, no study has evaluated whether there are differences in the seroprevalence of Chagas disease in rural and urban indigenous communities to assess the influence of the different risk factors.

The aim of this study was to assess the prevalence of Chagas disease in urban and rural moqoit communities, one of the main ethnic indigenous groups in the south of Gran Chaco, and to determine whether the prevalence of the disease differs between the two groups. 

## METHODS

### Study area

The study was conducted in indigenous communities from the south of the Gran Chaco ecoregion in Santa Fe Province, Argentina, between October 2012 and September 2015. In this province, the Instituto Provincial de Aborígenes Santafesinos (Provincial Institute of the Indigenous communities of Santa Fe) has recorded 46 indigenous communities^11^, 13 of which live in the Gran Chaco region. Ten of these communities belong to the Moqoit ethnic group, and three belong to the qom ethnic group. Six of the ten moqoit communities were selected as a result of the previous contacts we had with them. Two of these six communities live within cities (urban communities), and four are remote rural communities. An urban community was considered to have more than 2000 inhabitants, as defined by the National Institute of Statistics and Census of Argentina^12^.

### Study design

This observational, cross-sectional, descriptive study was conducted through convenience sampling of volunteers aged>1 year. For the recruitment of volunteers, both health leaders and community leaders (*cacique*s) were contacted to explain the methodology and scope of the project and obtain informed consent from participants. Health leaders invited volunteers to participate during home visits, whereas community leaders invited volunteers during community meetings.

Following biosecurity standards for the management of biological samples, blood samples were taken from volunteers by venous puncture, either in community meeting rooms or at their homes, in case they were unable to travel. Due to the type of recruitment, the reasons for rejecting informed consent were not recorded. Blood samples were transported to the laboratory of the National Endemia Research Center of the School of Biochemistry and Biological Sciences of the Universidad Nacional del Litoral, Santa Fe, Argentina, for processing and analyzed according to the Guía para la Atención del Infectado por *Trypanosoma cruzi* del Ministerio de Salud de la Nación (Guidelines for the assistance to people infected by *Trypanosoma cruzi* of the Argentine Ministry of Health). All blood samples were analyzed using indirect hemagglutination (Chagatest HAI Wienerlab®) and recombinant enzyme-linked immunosorbent assays (Chagatest ELISA Wienerlab®). In case of mismatch, an indirect immunofluorescence test was also performed^13^. The results were considered positive or negative based on the concordance between the two tests. 

### Statistical analysis

The variables studied were the serology results of Chagas disease, residents of urban or rural areas, age, and sex.

The data were incorporated into an Excel® database and analyzed using Epi-Info® 7.2.1. and InfoStat®. The continuous variable (age) was analyzed using the Mann-Whitney U test and summarized by the median value due to its non-Gaussian distribution and dichotomized to compare different groups by means of the prevalence ratio. The significance level was set at 5%. For the purpose of the analysis, the volunteers were divided into three age groups: < 19 years, 19-50 years, and >50 years. This division into age groups was based on the recommendations of the Argentine Ministry of Health regarding treatment of chronic patients: treat patients up to 19 years of age, probably treat patients between 20 and 50 years, and probably not treat patients over 50 years old. In addition, the seroprevalence of Chagas disease in women of childbearing age (15-44 years) has been described. The prevalence ratio was calculated using the corresponding confidence interval. 

### Ethical considerations

Participants were informed about the risks and benefits of blood collection. For volunteers between the ages of 1 and 18 years, informed consent to participate was provided by a responsible adult, whereas for those over 18 years, consent was given by the participants themselves. The results were then provided to each participant and to representative institutions of the Ministry of Health of the Province (Provincial Chagas Program and Local Health Centers) to coordinate and ensure accessibility to clinical follow-up and treatment. The procedures followed were in accordance with the ethical standards of the Personal Data Protection Law of Argentina (N° 25326) and the principles of the Declaration of Helsinki, 1964, as revised in 1975, 1983, 1989, 1996, and 2000.This project was evaluated and approved by the Ethics and Security Advisory Committee in Research of the School of Biochemistry and Biological Sciences of the Universidad Nacional del Litoral, Santa Fe, Argentina (Act No. 03.12).

## RESULTS

The six moqoit communities studied had an estimated population of 1543 inhabitants^14^. In total, 702 volunteers from this estimated population (45.50 %) were included in the study. Of these volunteers, 32.34% (227/702) belonged to two urban communities and 67.67% (475/702) to four rural communities. Regarding sex, 55.70% (391/702) were women and 44.30% (311/702) were men, with an age range between 1 and 94 years old and a median of 11 (15 in urban areas, 10 in rural areas).

The seroprevalence of Chagas disease was 9.26% (65/702), increasing from 1.94% in volunteers younger than 19 years old to 54.29% in those over 50 years old; 33.50% (131/391) of the participating women were of childbearing age (15 to 44 years of age)^15^, and 18.32% of these (24/131) women were seropositive for the infection. The results are summarized in Table 1.

**TABLE 1:** Sex, Age range and Serology for Chagas disease in urban and rural moqoit communities of the Gran Chaco of Santa Fe province, Argentina.

Age range	Sex				Type of community				Serology		Total
									Positive	% (95% Confidence Interval)	
<19 years	273	51.90	253	48.10	127	24.14	399	75.86	10	1.90	526
		(47.63-56.14)		(43.86-52.37)		(20.68-27.98)		(72.02-79.32)		(1.04-3.46)	
19-50 years	98	69.50	43	30.50	70	49.65	71	50.35	36	25.53	141
		(61.20-76.97)		(23.03-38.80)		(41.12-58.18)		(41.82-58.88)		(18.57-33.55)	
>50 years	20	57.14	15	57.14	30	85.71	5	14.29	19	54.29	35
		(39.35-73.68)		(26.32-60.65)		(69.74-95.19)		(4.81-30.26)		(36.65-71.17)	
Total	391	55.0	311	44.30	227	32.34	475	67.66	65	9.26	702
		(52.00-59.33)		(40.67-48.00)		(29.98-35.88)		(64.12-71.02)		(7.33-11.63)	

According to the calculated prevalence ratio, the seroprevalence of Chagas disease was 6.4(95% confidence interval (CI): 3.73-11.02) times higher in the urban population than in the rural population (Table 2).

**TABLE 2:** Association between serology for Chagas disease in moqoit communities and condition of living in a rural area in Gran Chaco of Santa Fe province, according to age ranges.

Age range	Community	Serology for Chagas				Prevalence Ratio (95% Confidence Interval)
		Positive	Negative	Total	Seroprevalence	
<19 years	Urban	5	122	127	3.93	3.14 (0.92-10.68)
	Rural	5	394	399	1.25	
19-50 years	Urban	25	45	70	35.71	2.31 (1.23-4.32)
	Rural	11	60	71	15.49	
>50 years	Urban	19	11	30	63.33	Non-defined
	Rural	0	5	5	0	
Total	Urban	49	178	227	21.5	6.41 (3.73-11.02)
	Rural	16	459	475	3.37	

**Reference group:** Community Rural.

## DISCUSSION

The seroprevalence of Chagas disease found in the indigenous communities studied was higher than the level estimated in the total population of the Santa Fe province (4%)^16^ ^,^ ^17^. These data coincide with the increased seroprevalence found in other studies related to indigenous communities of Gran Chaco compared with the general population^9^ ^,^ ^18^. As Chagas disease is an endemic disease strongly related to socio-sanitary conditions, the results would confirm that the indigenous peoples of this eco-region are more exposed to this neglected disease than the rest of the population. 

Living in an urban community was significantly associated with positive Chagas disease serology in all age groups. High discrepancy in seroprevalence between rural and urban areas in those older than 50 years was not discussed due to the small number of volunteers in this age range in rural communities.

The high seroprevalence found in women of childbearing age highlights the importance of campaigns for the detection and early treatment of congenitally infected newborns, as congenital transmission has been shown to be of increasing epidemiological importance^10^ ^,^ ^19^ ^,^ ^20^. The specific etiological treatment for women of childbearing potential has also been identified as the only effective form of prevention of congenital transmission^21^ ^-^ ^23^. In line with other studies describing the "urbanization" of the disease due to the immigration of people from endemic areas^6^ ^-^ ^8^, our results showed high seroprevalence in urban indigenous populations.

In 2012, the Santa Fe province was certified by the Pan American Health Organization as being free of vector transmission; therefore, the main route of transmission was congenital. The urbanization of indigenous communities in Santa Fe province is a fairly recent phenomenon, mainly occurring in the second half of the 20th century^24^. In this process, those who migrated the most were those with the worst socio-sanitary conditions in their places of origin, which also exposed them differentially to Chagas disease. For these groups, currently over 50 years old, congenital transmission could have caused the higher seroprevalence in urban areas compared torural areas for other age ranges.

Given the fact that the paradigm shift in the transmission of Chagas disease has occurred in recent years, from the vector to the congenital pathway and from the rural to the urban environment, it is necessary to expand public health policies, directing activities not only towards vector control in rural areas, but also to the timely diagnosis and treatment of mothers and children in both urban and rural areas. Likewise, such public policies should be accompanied by a more inclusive health system capable of ensuring accessibility to the diagnosis, treatment, and control of infections, especially for the most vulnerable social groups.

In conclusion, the seroprevalence found in the moqoit community was higher than the level estimated for the general population of the same region, with a greater impact in urban areas than in rural areas. The urbanization of groups of people with poor socio-sanitary conditions in the second half of the 20th century could have caused this higher seroprevalence of Chagas disease.

## References

[1] Pérez-Molina J, Molina I (2018). Chagas disease. Lancet.

[2] Crocco L, Nattero J, López A, Cardozo M, Soria C, Ortiz V (2019). Factors associated with the presence of triatomines in rural areas of south Argentine Chaco. Rev Soc Bras Med Trop.

[3] Benedictto M, Gómez-Valencia B, Torrella S (2019). Structural and functional characterization of the dry forest in central Argentine Chaco. Madera y Bosques.

[4] Colussi C, Stafuza M, Denner S, Nepote M, Mendicino D (2016). Epidemiología de la enfermedad de Chagas en comunidades Mocovíes y Criollas en el sur del Chaco Argentino. Salud Publica Mex.

[5] Lucero R, Bruses B, Cura C, Formichelli L, Juiz N, Fernández G (2016). Chagas' disease in Aboriginal and Creole communities from the Gran Chaco Region of Argentina: Seroprevalence and molecular parasitological characterization. Infect Genet Evol.

[6] Beloscar J, Rosillo I, Lioi S, Pituelli N, Corbera M, Turco M (2007). Migración aborigen y urbanización de la enfermedad de Chagas. Rev Fed Argent Cardiol.

[7] Mendicino D, Streiger M, Del Barco M, Fabbro D, Bizai ML (2010). Chagasic infection and related epidemiological antecedents in a low endemicity area of Argentina. Enf Emerg.

[8] Moscatelli G, Berenstein A, Tarlovsky A, Siniawski S, Biancardi M, Ballering G (2015). Urban Chagas disease in children and women in primary care centres in Buenos Aires, Argentina. Mem Inst Oswaldo Cruz.

[9] Fernández M, Gaspe MS, Gürtler R (2019). Inequalities in the social determinants of health and Chagas disease transmission risk in indigenous and creole households in the Argentine Chaco. Parasit Vectors.

[10] Danesi E, Fabbro DL, Segura EL, Sosa-Estani S (2020). Higher congenital transmission rate of Trypanosomacruzi associated with family history of congenital transmission. Rev Soc Bras Med Trop.

[11] Ministerio de Justicia y Derechos Humanos, Gobierno de la Provincia de Santa Fe (2014). Listado de comunidades aborígenes.

[12] Instituto Nacional de Estadísticas y Censos de Argentina (2010). Glosario del Censo Poblacional.

[13] Streiger M, Bovero N (1980). Indirect immunofluorescence reaction for the diagnosis of Chagas disease. Medicina.

[14] Instituto Nacional de Estadística y Censos (2010). Encuesta Complementaria de Pueblos Indígenas. 2005.

[15] Organización Mundial de la Salud. Centro de prensa (2013). Salud de la Mujer.

[16] Spillmann C, Burrone S, Coto H (2013). Análisis de la situación epidemiológica de la enfermedad de Chagas en Argentina: avances en el control, 2012. Rev Argent Salud Publica.

[17] Mendicino D, Colussi C, Stafuza M, Manattini S, Montemagiore S, Nepote M (2019). Seroprevalencia de Chagas en mayores de 14 años de áreas rurales del Chaco Santafesino. Rev Fac Cien Med Univ Nac Cordoba.

[18] Moretti E, Castro I, Franceschi C, Basso B (2010). Chagas disease: serological and electrocardiographic studies in Wichi and Creole communities of Misión Nueva Pompeya, Chaco, Argentina. Mem Inst Oswaldo Cruz.

[19] Dias N, Carvalho BD, Nitz N, Hagström L, Vital T, Hecht M (2019). Congenital Chagas disease: alert of research negligence. Rev Soc Bras Med Trop.

[20] Danesi E, Codebo MO, Sosa-Estani S (2019). Transmisión congénita de Trypanosoma cruzi. Argentina 2002-2014.. Medicina.

[21] Fabbro D, Danesi E, Olivera V, Codebó M, Denner S, Heredia C (2014). Trypanocide treatment of women infected with Trypanosoma cruzi and its effect on preventing congenital Chagas. PLoS Negl Trop Diseases.

[22] Moscatelli G, Moroni S, García-Bournissen F, Ballering G, Bisio M, Freilij H (2015). Prevention of congenital Chagas through treatment of girls and women of childbearing age. Mem Inst Oswaldo Cruz.

[23] Murcia L, Simón M, Carrilero B, Roig M, Segovia M (2017). Treatment of infected women of childbearing age prevents congenital Trypanosoma cruzi infection by eliminating the parasitemia detected by PCR. J Infect Dis.

[24] Gomitolo M, Cabré P, Cardozo L (2020). Dispositivos contemporáneos de visibilización, reconocimiento y registro de pueblos originarios en la Provincia de Santa Fe. Revista Pampa.

